# High-Throughput Chemical Screening for Antivirulence Developmental Phenotypes in Trypanosoma brucei

**DOI:** 10.1128/EC.00335-13

**Published:** 2014-03

**Authors:** Paula MacGregor, Alasdair Ivens, Steven Shave, Iain Collie, David Gray, Manfred Auer, Keith R. Matthews

**Affiliations:** a Centre for Immunity, Infection and Evolution, Institute for Immunology and Infection Research, School of Biological Sciences, University of Edinburgh, Edinburgh, United Kingdom; b Centre for Translational and Chemical Biology, School of Biological Sciences and School of Biomedical Sciences, University of Edinburgh, Edinburgh, United Kingdom; c Biological Chemistry and Drug Discovery, College of Life Sciences, University of Dundee, Dundee, United Kingdom

## Abstract

In the bloodstream of mammalian hosts, the sleeping sickness parasite, Trypanosoma brucei, exists as a proliferative slender form or a nonproliferative, transmissible, stumpy form. The transition between these developmental forms is controlled by a density-dependent mechanism that is important for the parasite's infection dynamics, immune evasion via ordered antigenic variation, and disease transmissibility. However, stumpy formation has been lost in most laboratory-adapted trypanosome lines, generating monomorphic parasites that proliferate uncontrolled as slender forms *in vitro* and *in vivo*. Nonetheless, these forms are readily amenable to cell culture and high-throughput screening for trypanocidal lead compounds. Here, we have developed and exploited a high-throughput screen for developmental phenotypes using a transgenic monomorphic cell line expressing a reporter under the regulation of gene control signals from the stumpy-specific molecule PAD1. Using a whole-cell fluorescence-based assay to screen over 6,000 small molecules from a kinase-focused compound library, small molecules able to activate stumpy-specific gene expression and proliferation arrest were assayed in a rapid assay format. Independent follow-up validation identified one hit able to induce modest, yet specific, changes in mRNA expression indicative of a partial differentiation to stumpy forms in monomorphs. Further, in pleomorphs this compound induced a stumpy-like phenotype, entailing growth arrest, morphological changes, PAD1 expression, and enhanced differentiation to procyclic forms. This not only provides a potential tool compound for the further understanding of stumpy formation but also demonstrates the use of high-throughput screening in the identification of compounds able to induce specific phenotypes, such as differentiation, in African trypanosomes.

## INTRODUCTION

Members of the order Kinetoplastida comprise a highly divergent group of eukaryotic organisms, with several representatives causing life-threatening and debilitating human diseases in a human-human or zoonotic transmission cycle. Trypanosoma brucei sp. is a blood-dwelling parasitic organism of this order, transmitted by tsetse flies, that causes both human and animal African trypanosomiasis (HAT and AAT, respectively) in sub-Saharan Africa. These diseases cause morbidity and then death when untreated in humans (1) and significantly contribute to poverty and restrict economic development in afflicted regions. Due to the poor safety and efficacy of current drugs, new treatments for African trypanosomiasis are urgently required (2, 3), and, consequently, considerable efforts are being made to search for new trypanocidal compounds. One approach has been through whole-cell-based high-throughput screening (4–10) where thousands of small molecules are rapidly analyzed for antiparasitic activity, identifying classes of lead compounds with potential for further development as antitrypanosomal drugs (6, 7, 9, 10). In addition to the use of high-throughput chemical inhibitor screens for the identification of compounds with antiparasitic properties, similar approaches have been used in Plasmodium falciparum and Toxoplasma gondii to search for small molecules able to induce specific cytological phenotypes (11–13), providing potential novel therapeutic approaches and tool compounds for biological research (14).

In the bloodstream of their mammalian host, African trypanosomes differentiate from the proliferative slender form to the transmissible stumpy form (15), a transition that is key to the within-host infection dynamics and transmissibility of the parasite (16). In naturally occurring pleomorphic cell lines, stumpy formation is triggered by parasite density, as sensed through the accumulation of an unidentified stumpy induction factor (SIF) (17). Although laboratory-adapted monomorphic bloodstream forms have lost the ability to differentiate to stumpy forms in response to population density *in vitro* or *in vivo*, these cell lines do have the capacity for differentiation to a “stumpy-like” state when treated with cell-permeable cyclic AMP (8-pCPT-cAMP) and, more potently, the hydrolysis products of these compounds and cell-permeable AMP, 8-pCPT-2′-*O*-methyl 5′-AMP (16–19). In response to these stimuli, monomorphic cells undergo cell cycle arrest, demonstrate an increased capacity for differentiation to the procyclic stage, and undergo changes in gene expression associated with stumpy formation (16–18). It has been hypothesized that cAMP analogues act to mimic the induction of stumpy formation, bypassing the initial step(s) of environmental density sensing and triggering at least part of the stumpy induction signaling cascade within the cell. Until recently, very little was known regarding the molecules involved in stumpy formation, with only negative regulators TbTOR4 kinase (Tb927.1.1930), which prevents stumpy formation in monomorphic slender forms (20), and two further inhibitory kinases, mitogen-activated protein kinase 5 (MAPK5) (Tb927.6.4220) (21) and zinc finger kinase (ZFK) (Tb927.11.9270) (22), having been identified. However, recently, promoters of stumpy formation have also been identified based on the selection of monomorphic RNA interference (RNAi) libraries for resistance to cell-permeable cAMP/AMP analogues, and these promoters have been further validated *in vivo* using pleomorphs for SIF responsiveness. Hence, a cohort of molecules representing many steps in the signal response leading to stumpy formation have now been identified, with small-molecule drivers of the stumpy-like response having been central to identification of the pathway components. Clearly, this knowledge of the stumpy induction pathway could also lead to novel therapeutic approaches since molecular inhibitors of stumpy formation could be targeted to induce premature development in the bloodstream, reducing parasite virulence or reducing abundance below a transmission threshold. Alternatively, molecular drivers of stumpy formation could be activated to achieve the same therapeutic outcome. Hence, compounds promoting developmental arrest in the parasite have value as biological tool compounds as well as offering novel approaches to disease control (20–22).

We have previously demonstrated that transgenic cell lines that utilize reporter genes (12) coupled to the 3′ untranslated region (UTR) of the PAD1 gene (Tb927.7.5930), a functional molecular marker for stumpy forms (23), can report on the response of monomorphic slender cells to conditions that promote the production of stumpy-like forms (16). Here, we have built on this system to develop a simple high-throughput assay for the detection of stumpy-like induction in monomorphic cell lines. This assay has been used to screen over 6,000 kinase-focused inhibitors for their ability to induce PAD1 3′ UTR-regulated reporter expression as a proxy for the induction of stumpy formation. This led to the identification of two structurally similar compounds that caused an unspecific increase in reporter expression as well as one chemically distinct compound that not only caused an upregulation of PAD1 mRNA expression but also generated consistent changes in mRNA expression for a small set of genes that are representative of partial stumpy formation in monomorphs and that also generate a stumpy-like phenotype in pleomorphic cell lines. This demonstrates the validity of a reporter screen for stumpy formation and of the use of high-throughput screening for the identification of small compounds that induce not only the cytocidal or cytostatic outcomes routinely analyzed but also specific phenotypic responses useful for the molecular analysis of trypanosome biology.

## MATERIALS AND METHODS

### Trypanosoma brucei cell lines and culturing.

Lister 427 cells were transfected with the pHD617 glucuronidase (GUS)-PAD1 3′ UTR reporter construct modified from pHD617 chloramphenicol acetyltransferase (CAT)-PAD1 3′ UTR (16, 24) to generate the cell line 427 GUS-PAD1 3′ UTR used for compound screening. The previously described (16) 427 CAT-PAD1 3′ UTR GUS-Const 3′ UTR cell line was utilized for follow-up analysis (16). Trypanosomes were grown in HMI-9 medium with 20% fetal calf serum (FCS) at 37°C in 5% CO_2_ (25).

### Differentiation experiments and assays of stumpy formation.

Differentiation of bloodstream forms to procyclic forms was induced by addition of 6 mM *cis*-aconitate and a temperature reduction from 37°C to 27°C. Differentiation capacity was determined by EP procyclin expression, measured by flow cytometry. Cell cycle analysis was carried out by 4′,6′-diamidino-2-phenylindole (DAPI) staining of fixed cells (100 ng/ml) and analysis via flow cytometry (26). For phase-contrast/DAPI microscopy, cells were fixed in ice-cold methanol and stained with 1 μg/ml DAPI. PAD1 protein expression was measured either by immunofluorescence microscopy with cells fixed for 10 min in 3% ice-cold paraformaldehyde (PFA) and stained using an anti-PAD1 antibody (23), by staining fixed cells for PAD1 protein and analyzing via flow cytometry, or by Western blot, carried out as previously described (23), with protein detected using Pierce ECL Western blotting substrate. 8-pCPT-cAMP was purchased from Sigma-Aldrich (United Kingdom).

### Compound screening.

The compound screening was carried out at the Drug Discovery Unit at the University of Dundee using a kinase-focused compound library from the Scottish Hit Discovery Facility. A kinase inhibitor library was chosen for two reasons: first, kinases play an important role in many cell processes, including stumpy formation (20–22), and, second, kinases have been proposed as good drug targets in trypanosomes (27–29). The 427 GUS-PAD1 3′ UTR cell line was plated into 96-well plates at 5 × 10^5^ cells/ml at 100 μl/well. Cells were treated for 48 h with test compounds at 50 μM in single cultures during primary screening and at 5.6 μM, 16.7 μM, and 50 μM in duplicate cultures during secondary screening in 0.5% (vol/vol) dimethyl sulfoxide (DMSO). This initial 50 μM concentration was chosen for primary screening based on standard screening procedures at the Drug Discovery Unit and because the positive control for induction of a stumpy-like form *in vitro* is routinely used at 100 μM 8-pCPT-cAMP (16) (Sigma, United Kingdom). Secondary screening was carried out in a titration series to assess hit potency although for tool compound development this is less important than phenotypic specificity. Screen performance was determined via internal controls (see Fig. 2), with a *Z*′ score (based on GUS enzyme assay) of 0.61 ± 0.13 for primary screening and 0.58 ± 0.12 for secondary screening (mean ± standard deviation). The purity and molecular mass of all hit compounds were determined by liquid chromatography-mass spectrometry.

GUS enzyme activity was measured via the addition of an equal volume of 4-methylumbelliferyl-β-glucopyranosiduronic acid (MUG; Sigma, United Kingdom) in lysis buffer (1 mM MUG, 0.82 M Tris-HCl, pH 8, 0.6% SDS, 0.3 mg/ml bovine serum albumin [BSA]) and measuring fluorescence at an excitation wavelength (λ_ex_) of 340 nm and emission wavelength (λ_em_) of 460 nm after a 2-h incubation. Hit identification was based on results from a GUS enzyme assay only; however, for a measure of cell growth, 10% (vol/vol) alamarBlue was also added to the cells, and fluorescence was measured at λ_ex_ of 530 nm and λ_em_ of 590 nm after a 4-h incubation (30). The alamarBlue assay was carried out immediately prior to the GUS assay, and no cross talk was observed. Fluorescence measured in GUS assays carried out during follow-up analysis was corrected for cell number. Addition of pCPT-cAMP, compound DDD00070762, or compound DDD00015314 to HMI-9 medium did not cause any increase in background fluorescence compared to DMSO-treated HMI-9 medium.

Validation analysis using a CAT-based reporter was also used. Here, CAT protein levels were determined by CAT enzyme-linked immunosorbent assay (ELISA; Roche) according to the manufacturer's instructions, with absorbance being measured using a BioTek ELx808 Absorbance Microplate Reader. Statistical analysis was carried out using Minitab, version 16, with data analyzed using general linear models (GLMs). Transformation of data was carried out to satisfy assumptions of normality. *P* values of less than 0.05 were considered significant.

### RNA sequencing.

RNA samples for analysis by high-throughput sequencing of RNA transcripts (RNA-Seq) were harvested from CAT-PAD1 3′ UTR GUS-Const 3′ UTR cells after treatment with 50 μM DDD00015314 or 0.5% (vol/vol) DMSO, in duplicate, for 24 h. Library construction and sequencing were carried out by BGI-Hong Kong using an Illumina TruSeq RNA Sample Preparation Kit. The quality of raw sequence data was assessed using FastQC (http://www.bioinformatics.babraham.ac.uk/projects/fastqc/). Paired-end sequences were aligned to the Trypanosoma brucei brucei genome (obtained from ftp.sanger.ac.uk/pub4/pathogens/Trypanosoma/brucei/Latest_Whole_Genome_Sequence/Tb927_WGS_24_08_2012/chromosomes/) using Bowtie2 (parameters, very-sensitive-local; version 2.0.2); the outputs were filtered to remove alignments flagged with the XS Sequence Alignment/Map (SAM) flag, yielding an alignment data set that included only reads that map to a single location in the reference genome. These data were subsequently sorted and indexed using samtools. The annotated Trypanosoma brucei brucei genome was viewed using Artemis software (http://www.sanger.ac.uk/resources/software/artemis/ and http://ukpmc.ac.uk/abstract/MED/11120685), and coding segment (CDS) region coordinates were extracted prior to conversion to bedtools bedfile format. Bedtools (version 2.15.0; parameters, multicov, -bams) was used to generate coverages for each CDS for each sample replicate. Reads per kilobase per million reads mapped (RPKM) values were calculated by dividing coverage by CDS size using only reads that mapped to a single location in the reference genome (RPKM-like). Statistical analyses of the two sample groups were undertaken in the R environment (www.R-project.org) using Bioconductor (www.bioconductor.org) packages. Differential expression was explored using linear models and empirical Bayes methods, using the limma package (31). RPKM-like values were offset by 1, logged, and quantile normalized prior to group-wise comparison. Postcomparison, data were filtered to remove loci whose normalized mean RPKM-like values were below the 20% quantile for all samples.

Meta-analysis of the log_2_ expression data from Capewell et al. (32) and Jensen et al. (33) required identification of overlapping gene sets, achievable using systematic gene names. However, as there have been many improvements in both the sequence and annotation of the Trypanosoma brucei brucei 927 genome, some loci had been renamed, with the previous names retained in their annotation. Using simple parsing scripts, a table of current and previous gene names was generated, thereby facilitating data set comparisons.

### Nucleotide sequence accession number.

RNA sequence data have been deposited in the NCBI Gene Expression Omnibus (GEO) repository under accession number GSE46483.

## RESULTS

### Validation of the reporter system.

The 3′ UTR of the PAD1 gene has been shown, at least in part, to control its stumpy-specific gene expression (16). By coupling the PAD1 3′ UTR to a reporter gene, it is possible to monitor the increase in expression of reporter activity as an indicator of stumpy-like formation or the induction of stumpy-enriched mRNA expression. This method has been used previously for the analysis of known chemical inducers of stumpy-like formation via a CAT reporter gene (16), where treatment with cell-permeable cAMP (8-pCPT-cAMP) or a cAMP analogue (8-pCPT-2′-*O*-methyl-cAMP) caused an increase in CAT mRNA and protein expression as well as cell growth arrest and more efficient *in vitro* differentiation to procyclic forms after exposure to 6 mM *cis*-aconitate. Hence, these analogues drive the expression of several characteristics associated with physiological stumpy formation, there being an intersection between the molecules responsible for response to these analogues and the response to SIF itself (26). Nonetheless, it should be noted that while cell-permeable cAMP is able to induce upregulation of protein levels of reporter genes coupled to the PAD1 3′ UTR in monomorphic cell lines, it does not induce upregulation of the PAD1 protein itself (see Fig. S1 in the supplemental material), suggesting that there are additional levels of expression control operating on PAD1 in these cells.

### High-throughput screening for reporter activation.

**FIG 1:**
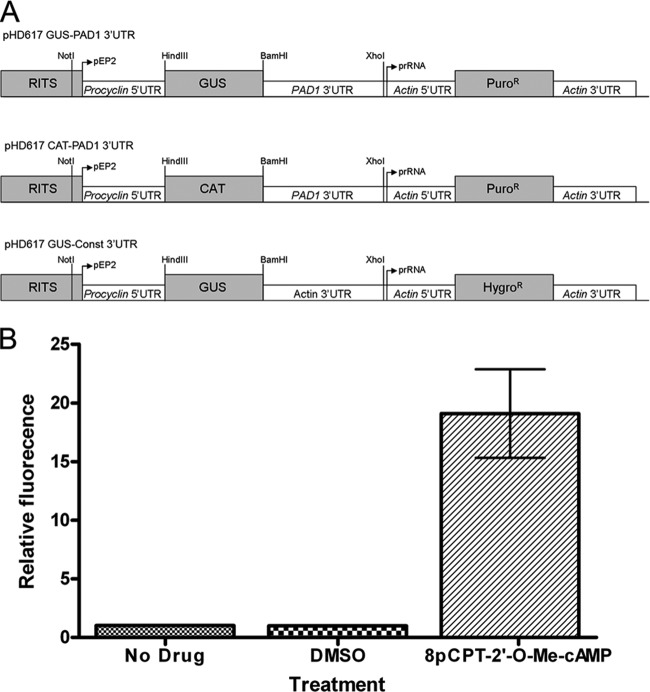
Regulation of GUS reporter gene expression by the PAD1 3′ UTR. (A) Schematic representation of the pHD617 GUS-PAD1 3′ UTR reporter construct used to create the 427 GUS-PAD1 3′ UTR cell line used for compound screening. This vector was modified from those described in MacGregor et al. and Biebinger et al. (16, 24). Also shown are the pHD617 CAT-PAD1 3′ UTR and pHD617 GUS-Const 3′ UTR reporter constructs previously used to create the 427 CAT-PAD1 3′ UTR GUS-Const 3′ UTR cells (16), which are utilized in this study for follow-up analysis. The vector integrates into the ribosomal spacer locus via a ribosomal integration site sequence (RITS). (B) Monomorphic bloodstream form T. brucei cells transfected with the pHD617 GUS-PAD1 3′ UTR construct (427 GUS-PAD1 3′ UTR cells) showed an increase in GUS reporter activity when treated with 10 μM 8-pCPT-2′-*O*-Me-cAMP. Treatment with 0.5% (vol/vol) DMSO had no effect on reporter activity. Error bars represent standard errors of the means (*n* = 3).Puro, puromycin; Hygro, hygromycin.

For the purposes of high-throughput screening, it was necessary to use a reporter that could be monitored quickly and cost effectively on a large scale. Hence, a β-glucuronidase reporter able to convert a nonfluorescent substrate into a fluorescent product was selected such that reporter activity could be measured in a rapid, simple, and economical assay. For the assay, a substrate/lysis buffer was added to whole cells, and fluorescence was measured, providing compatibility with a 96-well plate format. An expression vector was created that coupled the GUS reporter gene to the PAD1 3′ UTR (Fig. 1A), and this was transfected into T. brucei s427 monomorphic cells to create the 427 GUS-PAD1 3′ UTR cell line. Upon induction with 8-pCPT-2′-*O*-Me-cAMP (16, 18), these cells upregulated GUS reporter activity, confirming their ability to respond appropriately in the phenotypic screen (Fig. 1B). This also confirmed the ability of the PAD1 3′ UTR to respond to a stumpy trigger regardless of the reporter gene used.

**FIG 2:**
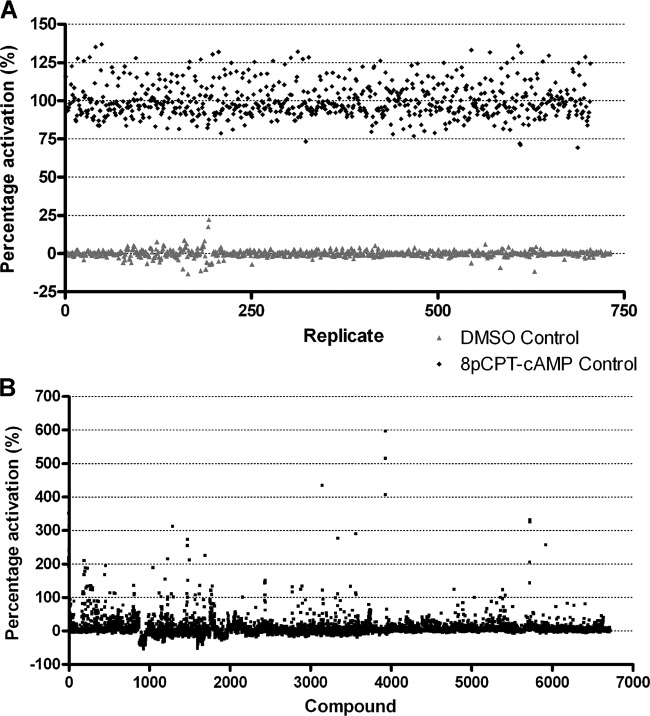
Scattergram of the results of primary screen. During primary screening, the 427 GUS-PAD1 3′ UTR cell line was treated with a 50 μM concentration of 6,764 compounds for 48 h. (A) In 96-well plates, eight wells on each plate contained 100 μM 8-pCPT-cAMP (positive control), and eight wells contained 0.5% (vol/vol) DMSO (negative control). The percent activation of the GUS reporter activity across all control wells during primary screening is shown, with the mean of all wells for 8-pCPT-cAMP treatment set at 100%. (B) Results of primary screening with 198 compounds; those showing >53% activation of GUS reporter activity were classified as possible hits.

**FIG 3:**
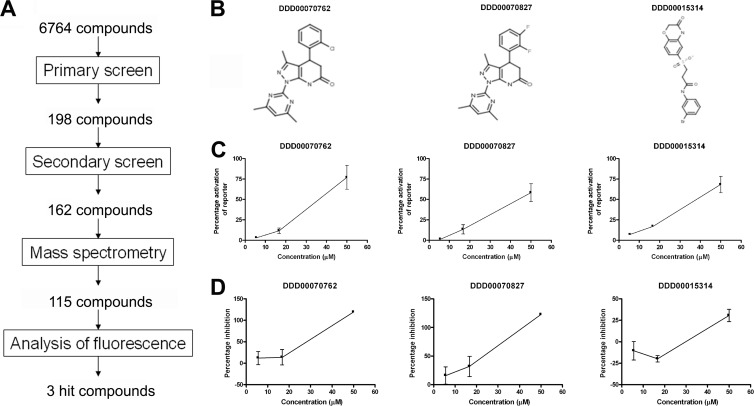
Three hit compounds were identified through screening for the ability to induce stumpy-specific reporter gene expression. (A) Overview of workflow used to identify three hit compounds from a library of 6,764 small molecules. (B) Structures of three hit compounds. (C) Percent GUS reporter activity after 48 h of treatment as measured during secondary screening for each of the three hit compounds at 5.6 μM, 16.7 μM, and 50 μM, where 100% activation is that caused by 100 μM 8-pCPT-cAMP. (D) Percent inhibition of alamarBlue activity after 48 h of treatment as measured during secondary screening for each of the three hit compounds at 5.6 μM, 16.7 μM, and 50 μM, where 100% inhibition is that caused by 100 μM 8-pCPT-cAMP. Data points represent the mean of two replicates, with error bars representing the range.

The 427 GUS-PAD1 3′ UTR cell line was used to carry out a pilot screen of small molecules with the aim of identifying novel chemical inducers of stumpy formation. The screen was carried out at the Drug Discovery Unit at the University of Dundee using a kinase inhibitor-focused small-molecule library composed of 6,764 molecules (Fig. 2), according to the work flow outlined in Fig. 3A. First, in a primary screen, 100 μl of 5 × 10^5^ cells/well of the 427 GUS-PAD1 3′ UTR cell line was incubated with a 50 μM concentration of each of the compounds from the library for 48 h in 96-well plates (Fig. 2). Prior to monitoring for GUS reporter activity, an alamarBlue assay (30) was carried out in order to provide a simple measure of cell proliferation; as stumpy formation is associated with cell cycle arrest (15), it was expected that any compound causing differentiation would also cause a decrease in proliferation of the population (data not shown). On all plates, wells treated with 100 μM 8-pCPT-cAMP were included as a positive control and as a benchmark for reporter activation (16, 17), with activation of the reporter and inhibition of cell proliferation induced by the 8-pCPT-cAMP set at 100% change. Given that this reports on a developmental transition (as opposed to enzymatic activity alone), physiologically relevant hit compounds would not be expected to generate a response significantly greater than that of the positive control (Fig. 2A). As such, compounds able to generate >53% GUS reporter activity, as determined through primary screening (Fig. 2B), were selected for secondary screening (mean *Z*′, 0.61) (Fig. 2A). This cutoff was selected to include the top ∼200 hits, a number suitable for secondary screening.

The selected compounds were next subjected to secondary screening whereby each compound was tested in duplicate at 5.6 μM, 16.7 μM, and 50 μM (see Data Set S1 in the supplemental material). From secondary screening, 162 compounds generated fluorescence with a mean of >53% at 50 μM, providing a signal confirmation rate of the assay of 81.8% (mean *Z*′, 0.58). All 162 compounds were examined via mass spectrometry to confirm purity and identity. Compounds that failed this analysis were removed, leaving 115 compounds. Since many of these generated signals far greater than 8-pCPT-cAMP, it was anticipated that biologically irrelevant hits would comprise a significant proportion of these. Therefore, these 115 compounds were tested for autofluorescence in the absence of trypanosomes. This step determined that 112 of the 115 compounds exhibited autofluorescence under the conditions used in the screen (λ_ex_ of 340 nm and λ_em_ of 460 nm) and, hence, likely represented false-positive results (see Data Set S1). To confirm that this group did not contain a significant proportion of biologically active autofluorescent compounds, a small selection of these 112 compounds were analyzed for their ability to induce stumpy reporter activation using an independent, nonfluorescent CAT reporter-based assay, with each exhibiting no activity although some of the compounds did affect cell growth (data not shown; see Fig. S2 in the supplemental material). Thus, to stringently select for compounds that represent biologically relevant hits with respect to the phenotypic screen used, all 112 autofluorescent compounds were excluded from further analysis, identifying three hits from a primary screen of 6,764 molecules (Fig. 3B). This apparently low hit rate of 0.04% highlights the selectivity of the screen for a differentiation-specific response; a far higher number of compounds (37% in the primary screen) caused trypanocidal or trypanostatic growth effects (as measured by alamarBlue assay), probably through multiple specific and nonspecific cellular mechanisms.

### Reporter and phenotypic confirmation of screen outputs.

Relative to 100 μM 8-pCPT-cAMP, the three hit compounds identified were able to activate the GUS reporter gene at levels between 58% and 77% at 50 μM (Fig. 3C). Each compound also caused a decrease in alamarBlue activation, indicative of a reduction in population growth of between 31% and 123%, where 100% inhibition is that caused by 100 μM 8-pCPT-cAMP (Fig. 3D). Of the three compounds, DDD00070762 and DDD00070827 were structurally related (Fig. 3B), and the third was chemically distinct.

**FIG 4:**
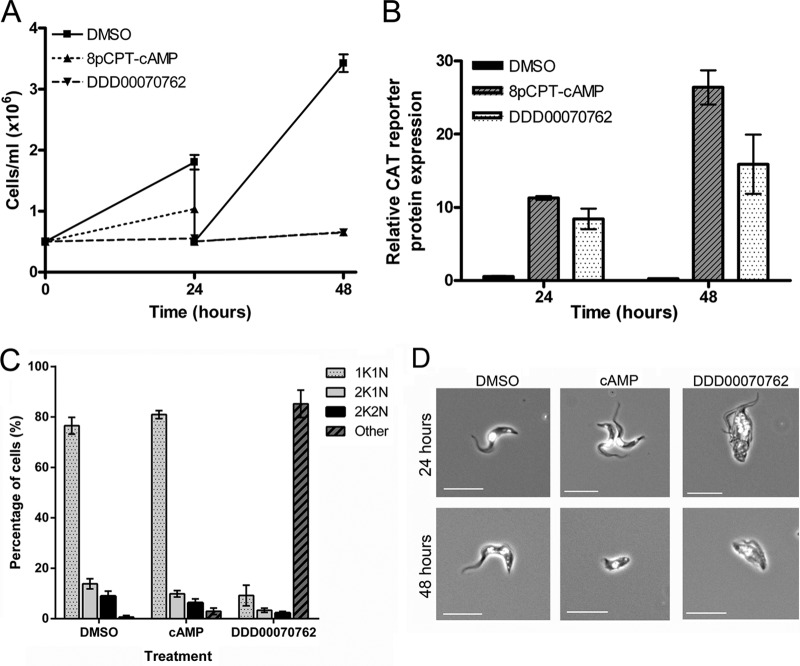
Compound DDD00070762 causes upregulation of stumpy reporter gene expression but not stumpy formation. 427 CAT-PAD1 3′ UTR GUS-Const 3′ UTR cells were treated with 50 μM DDD00070762. Negative-control cells were treated with 0.5% (vol/vol) DMSO; positive-control cells were treated with 100 μM 8-pCPT-cAMP. (A) Population growth was monitored at 24 and 48 h. Cells treated with DDD00070762 did not grow after 24 or 48 h. (B) CAT reporter gene expression, under the control of the PAD1 3′ UTR, increased after 24 and 48 h of treatment. GUS reporter gene expression, under the control of a constitutive 3′ UTR, showed variable expression levels between experiments after treatment (data not shown). (C) After 24 h of treatment, >80% of cells had an abnormal composition of DNA-containing organelles (nucleus [N] and kinetoplast [K]), from 250 cells per sample counted, in contrast with cells treated with 100 μM 8-pCPT-cAMP, where <5% of cells had aberrant DNA content after 24 h. (D) Examples of cells treated with DMSO, 8-pCPT-cAMP, or DDD00070762 for 24 h and 48 h are shown. Cells were stained with DAPI. Scale bar, 10 μm. Error bars in all graphs represent standard errors of the means (*n* = 3).

Of the two related compounds identified, compound DDD00070762 was chosen for validation and follow-up analysis. This compound demonstrated a 77% activation level of the GUS reporter activity during screening (Fig. 3C) and also caused reduced population growth as measured by an alamarBlue assay (Fig. 3D). For validation, 427 CAT-PAD1 3′ UTR GUS-Const 3′ UTR cells were used that have the CAT reporter gene under PAD1 3′ UTR control linked to a constitutively expressed GUS reporter (this provides a measure of constitutive gene expression in the presence of the compound in addition to stumpy-specific expression changes within the same cell line) (16). Treatment of these cells with 50 μM compound DDD00070762 stopped population growth within 24 h (Fig. 4A) and caused upregulation of CAT reporter gene expression after 24 and 48 h (Fig. 4B). This result confirmed that DDD00070762 promoted stumpy-specific reporter gene expression in an independent, non-fluorescence-based assay, validating the results of the high-throughput screen. It was also observed, however, that the control GUS reporter gene expression was highly variable between analyses (data not shown) and that within 24 h of treatment, >80% of the cells had become multinucleated (Fig. 4C). This contrasted with the small number of cells (<5%) that showed aberrant cell cycle types after 24 h of 8-pCPT-cAMP treatment, where multinucleated cells were not frequently observed. Indeed, while 8-pCPT-cAMP treatment causes monomorphic cells to display a somewhat atypical morphology after 48 h (Fig. 4D), this was distinct from the misshapen multinucleated cells that arose after only 24 h of DDD00070762 treatment. Hence, although the stumpy-linked reporter gene was upregulated by DDD00070762, this was not associated with other hallmarks of a physiological differentiation to stumpy-like forms. Rather, the compound appeared to cause considerable cell cycle and morphological defects unrelated to stumpy formation, and therefore DDD00070762 and the structurally related DDD00070827 were excluded from further analysis.

### DDD00015314 drives stumpy-enriched gene expression.

**FIG 5:**
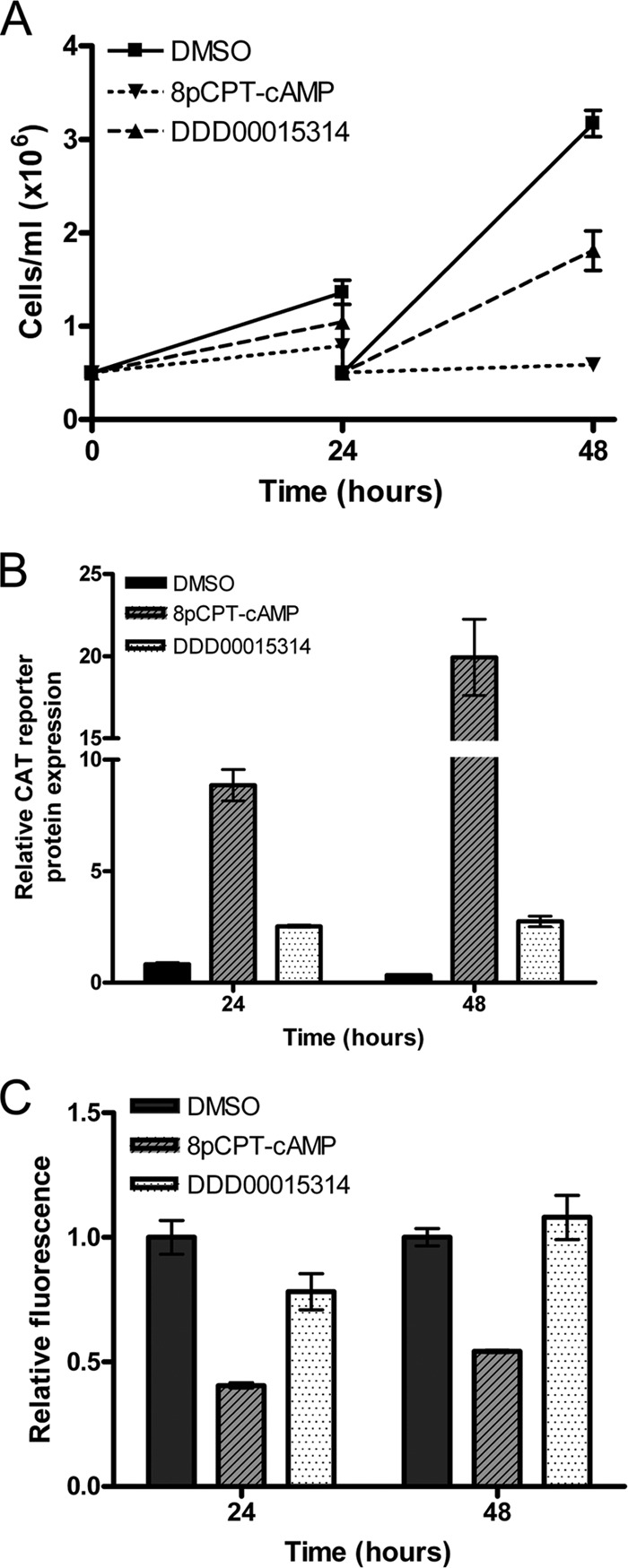
Compound DDD00015314 specifically causes a modest upregulation of stumpy-specific reporter gene expression without causing upregulation of constitutive reporter gene expression in monomorphic cells. 427 CAT-PAD1 3′ UTR GUS-Const 3′ UTR cells were treated with 50 μM DDD00015314. Negative-control cells were treated with 0.5% (vol/vol) DMSO; positive-control cells were treated with 100 μM 8-pCPT-cAMP. (A) Population growth was monitored at 24 and 48 h. Cells treated with DDD00015314 showed reduced growth compared to DMSO-treated controls. (B) CAT reporter gene expression, under the control of the PAD1 3′ UTR, increased after 24 and 48 h of treatment. (C) GUS reporter gene expression, under the control of a constitutive 3′ UTR, was slightly reduced after 24 h of treatment with DDD00015314; however, at 48 h GUS reporter gene expression was again equivalent to that of DMSO-treated controls. Error bars in all graphs represent standard errors of the means (*n* = 3).

Compound DDD00015314, *N*-(3-bromophenyl)-3-[(3-oxo-4H-1,4-benzoxazin-6-yl)sulfonyl] propanamide, did not show any structural similarities to either of the other two hits from the screen (Fig. 3B). This compound demonstrated a 68% activation of the GUS reporter activity during screening (Fig. 3C) and also caused a modest reduction in population growth, as measured by the alamarBlue assay (Fig. 3D). For validation, 427 CAT-PAD1 3′ UTR GUS-Const 3′ UTR cells were treated with a 50 μM concentration of the compound. As in the primary screen, this caused a modest reduction in population growth (Fig. 5A) to 43.6% of that of negative controls over 48 h. Also, a 2.5-fold increase in stumpy-specific reporter gene expression was reproducibly observed after 24 h of treatment (Fig. 5B) (DMSO-treated cells versus DDD00015314-treated cells at 24 h, *F*_1_,_4_ = 104.51, *P* = 0.001), which was not accompanied by any corresponding increase in the constitutive GUS reporter (Fig. 5C) (DMSO-treated cells versus DDD00015314-treated cells at 24 h, *F*_1_,_4_ = 4.92, *P* = 0.091). This is distinct from 8-pCPT-cAMP treatment, which results in reduced levels of GUS reporter expression, likely reflecting cellular quiescence of the treated parasites (34). This result demonstrated that DDD00015314 generated specific activation of the stumpy reporter although no morphological changes in the treated cells were observed by microscopy (data not shown). Hence, the overall response appeared to be less robust than with 8-pCPT-cAMP, but the compound nonetheless generated specific and reproducible activation of the reporter expression in monomorphic cells.

The specific increase in stumpy reporter gene expression induced by DDD00015314 suggested that the compound promoted some aspects of development to stumpy forms or stumpy-enriched gene expression. To investigate this, transcriptome analysis via RNA-Seq was carried out on treated and control cells to monitor global changes in gene expression in response to compound exposure. RNA samples were harvested from cells after treatment, in duplicate, for 24 h with 50 μM DDD00015314 or 0.5% (vol/vol) DMSO. The resulting total RNA was then subjected to RNA-Seq analysis following Illumina TruSeq RNA Sample Preparation (BGI, Hong Kong). Over 13 million 90-bp reads were generated per sample, with a quality score of 20 (Q20; i.e., error rate of ≤1%) in >95.5% of all samples. Raw sequence data were aligned to the Trypanosoma brucei brucei 927 genome, and reads per kilobase per million mapped reads (RPKM) were calculated for each CDS for each sample replicate, including only reads that map to a single location in the reference genome.

**FIG 6:**
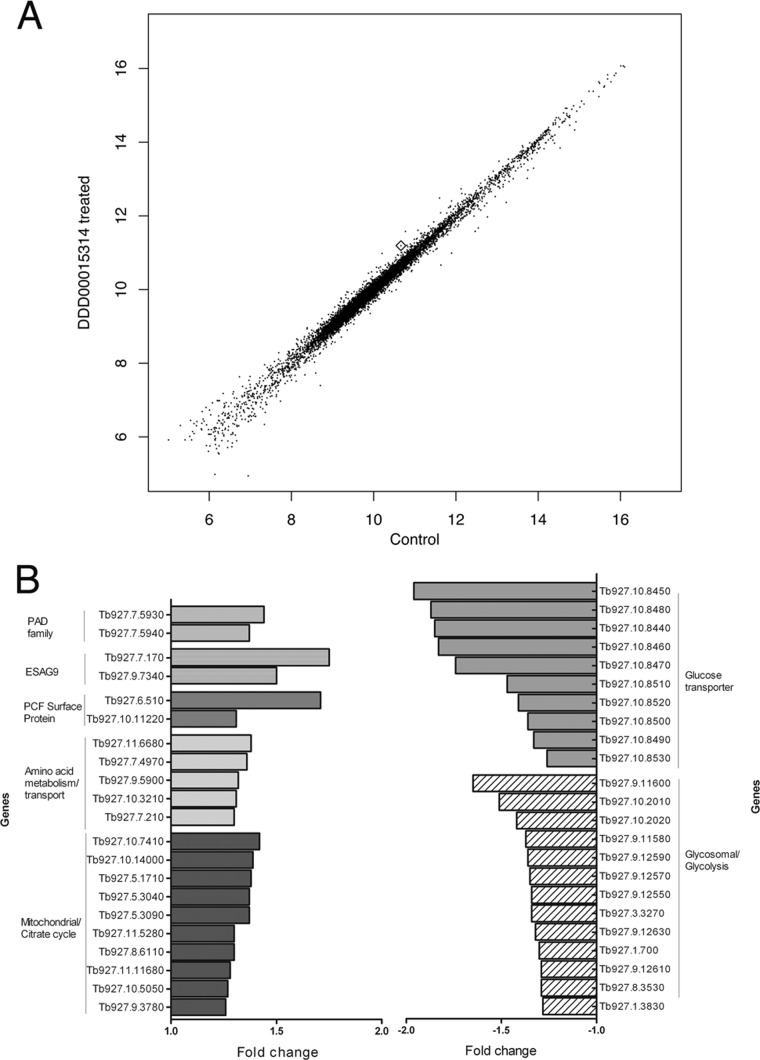
RNA-Seq on DDD00015314-treated cells reveals modest but specific changes in gene expression. (A) Scatter plot of log-normalized mean reads per kilobase per million mapped reads (RPKM) for control and DDD00015314-treated cells based on two replicates, with values below the 20% quartile removed. The PAD1 transcript is highlighted with a diamond. (B) Histogram showing absolute fold change of mRNA levels for selected genes that were up- and downregulated after DDD00015314 treatment. PCF, procyclic form.

Pairwise comparison between the populations treated with DDD00015314 or 0.5% (vol/vol) DMSO revealed a correlation of 0.995 (Fig. 6A), suggesting that treatment with 50 μM DDD00015314 caused little general perturbation of gene expression, despite the slowed growth of the treated cells, and revealing considerable consistency in the respective sample replicates. Nevertheless, 95 genes were identified as upregulated, and 71 genes were downregulated, with a ≥1.25-fold change after DDD00015314 treatment (see Data Set S2 in the supplemental material) and a *P* of <0.05 before adjustment for multiple testing, with no genes showing a significant change after adjustment for multiple testing. Table 1 shows a truncated list of genes up- and downregulated after DDD00015314 treatment, with all hypothetical (47/95 upregulated, 27/71 downregulated), variant surface glycoprotein (VSG; 3/95 upregulated, 6/71 downregulated), and retrotransposon hot spot protein (5/95 upregulated) genes removed for clarity.

**TABLE 1:** A truncated list of genes upregulated and downregulated in response to DDD00015314 treatment^*a*^

Gene	Description^*b*^	Group^*c*^	Fold change in expression	Fold change in slender vs stumpy forms according to:^*d*^	
				Capewell et al.	Jensen at al.
Upregulated genes					
Tb927.11.6680	Amino acid permease/transporter, putative	Amino acid metabolism ± transport	1.38	−1.26	−1.13
Tb927.7.4970	Glutamine synthetase, putative	Amino acid metabolism ± transport	1.36	4.27	1.31
Tb927.10.3210	Delta-1-pyrroline-5-carboxylate dehydrogenase, putative	Amino acid metabolism ± transport	1.31	1.25	1.54
Tb927.7.210	Proline dehydrogenase	Amino acid metabolism ± transport	1.30	1.14	1.74
Tb927.9.5900	Glutamate dehydrogenase	Amino acid metabolism ± transport	1.32	2.43	2.43
Tb927.7.170	ESAG9 protein, putative	ESAG/GRESAG	1.75	1.75	2.58
Tb927.9.7340	ESAG9 protein, putative	ESAG/GRESAG	1.50	NA	6.63
Tb927.8.7880	Receptor-type adenylate cyclase GRESAG 4 (pseudogene), putative	ESAG/GRESAG	1.38	−1.53	NA
Tb927.3.540	ESAG1 (pseudogene), putative	ESAG/GRESAG	1.27	2.75	NA
Tb927.9.16010	ESAG4 (pseudogene), putative	ESAG/GRESAG	1.33	7.35	NA
Tb927.8.2540	3-Ketoacyl-CoA thiolase, putative	Fatty acid metabolism	1.52	1.39	1.08
Tb927.11.16480	Enoyl-CoA hydratase/isomerase family protein, putative	Fatty acid metabolism	1.30	1.56	1.55
Tb927.8.6110	Hydroxymethylglutaryl-CoA synthase	Mitochondrial ± citrate cycle	1.30	1.88	1.09
Tb927.5.1710	Ribonucleoprotein p18, mitochondrial precursor, putative	Mitochondrial ± citrate cycle	1.38	−1.20	1.13
Tb927.9.3780	Putative mitochondrial pyruvate carrier 1 homolog	Mitochondrial ± citrate cycle	1.26	2.51	1.15
Tb927.11.5280	ATPase subunit 9, putative	Mitochondrial ± citrate cycle	1.30	1.70	1.20
Tb927.10.5050	Putative mitochondrial ATP synthase subunit	Mitochondrial ± citrate cycle	1.27	3.63	1.22
Tb927.10.14000	Aconitase	Mitochondrial ± citrate cycle	1.39	2.59	1.44
Tb927.5.3090	Putative mitochondrial ATP synthase subunit	Mitochondrial ± citrate cycle	1.37	1.79	1.68
Tb927.10.7410	Succinyl-CoA ligase (GDP-forming) beta-chain, putative	Mitochondrial ± citrate cycle	1.42	2.14	1.72
Tb927.5.3040	MIX protein	Mitochondrial ± citrate cycle	1.37	1.04	1.75
Tb927.11.11680	2-Oxoglutarate dehydrogenase E2 component, putative	Mitochondrial ± citrate cycle	1.28	1.06	2.28
**Tb927.7.5930**	** PAD1**	**PAD family**	**1.44**	**−1.17**	**1.23**
Tb927.7.5940	PAD2	PAD family	1.37	1.70	1.84
Tb927.8.6170	Transketolase, putative	Pentose phosphate pathway	1.32	3.15	2.16
Tb927.10.11220	PSSA-2	Procyclic form surface protein	1.31	5.78	1.55
Tb927.6.510	GPEET 2 procyclin precursor	Procyclic form surface protein	1.71	3.88	8.51
Tb927.7.3320	ATG12/APG12, putative	Protein/peptide degradation	1.25	1.06	−1.36
Tb927.11.2500	Carboxypeptidase, putative	Protein/peptide degradation	1.30	−1.26	−1.06
Tb927.10.12260	Cytosolic nonspecific dipeptidase, putative	Protein/peptide degradation	1.45	2.99	1.59
Tb927.4.3940	Calpain-like cysteine peptidase, putative	Protein/peptide degradation	1.33	1.87	1.71
Tb927.3.590	Adenosine transporter, putative	Purine metabolism ± transport	1.44	−2.21	−1.59
Tb927.11.15530	C-14 sterol reductase, putative	Steroid biosynthesis	1.28	2.56	1.56
Tb927.10.6910	Sterol methyltransferase, putative	Steroid biosynthesis	1.28	3.08	NA
Tb927.10.6950	Sterol 24-c-methyltransferase, putative	Steroid biosynthesis	1.25	4.07	NA
Tb927.10.12850	TTAGGG binding factor	Other	1.36	1.18	−1.07
Tb927.7.7110	LRRP, putative	Other	1.36	1.34	−1.01
Tb927.6.3130	Queuine tRNA-ribosyltransferase, putative	Other	1.30	4.64	1.16
Tb927.7.7180	LRRP, putative	Other	1.34	−1.02	NA
Tb927.8.4100	FLA1-binding protein (FLA1BP)	Other	1.26	1.47	NA
Downregulated genes					
Tb927.10.9480	ESAG3 protein, putative	ESAG/GRESAG	−1.34	Inf Up	1.02
Tb927.7.3250	ESAG6 protein, putative	ESAG/GRESAG	−1.47	−4.45	1.18
Tb927.7.3260	ESAG7 protein, putative	ESAG/GRESAG	−1.61	−2.98	1.30
Tb927.9.16700	ESAG3 (pseudogene), putative	ESAG/GRESAG	−1.49	1.33	NA
Tb927.10.8440	Glucose transporter 1B	Glucose transporter	−1.85	−2.39	−1.28
Tb927.10.8450	Glucose transporter 1E	Glucose transporter	−1.96	−3.01	−1.17
Tb927.10.8530	Glucose transporter 2A	Glucose transporter	−1.26	−1.27	1.08
Tb927.10.8510	Glucose transporter, putative	Glucose transporter	−1.47	−3.99	NA
Tb927.10.8500	Glucose transporter, putative	Glucose transporter	−1.36	−1.79	NA
Tb927.10.8480	Glucose transporter, putative	Glucose transporter	−1.87	−1.55	NA
Tb927.10.8460	Glucose transporter, putative	Glucose transporter	−1.83	−1.46	NA
Tb927.10.8520	Glucose transporter, putative	Glucose transporter	−1.41	−1.27	NA
Tb927.10.8470	Glucose transporter, putative	Glucose transporter	−1.74	−1.18	NA
Tb927.10.8490	Glucose transporter, putative	Glucose transporter	−1.33	3.70	NA
Tb927.1.700	PGKC	Glycosomal ± glycolysis/gluconeogenesis	−1.30	−5.38	−2.19
Tb927.3.3270	ATP-dependent phosphofructokinase	Glycosomal ± glycolysis/gluconeogenesis	−1.34	−6.47	−1.80
Tb927.9.11600	Gim5B protein	Glycosomal ± glycolysis/gluconeogenesis	−1.65	−2.96	−1.75
Tb927.9.11580	Gim5A protein	Glycosomal ± glycolysis/gluconeogenesis	−1.37	1.03	−1.61
Tb927.10.2010	Hexokinase (HK1)	Glycosomal ± glycolysis/gluconeogenesis	−1.51	−1.84	−1.51
Tb927.8.3530	Glycerol-3-phosphate dehydrogenase (NAD^+^), glycosomal	Glycosomal ± glycolysis/gluconeogenesis	−1.29	−2.31	−1.38
Tb927.1.3830	Glucose-6-phosphate isomerase, glycosomal	Glycosomal ± glycolysis/gluconeogenesis	−1.28	−4.85	−1.37
Tb927.9.12590	Glycerol kinase, glycosomal (glk1)	Glycosomal ± glycolysis/gluconeogenesis	−1.36	−3.06	−1.25
Tb927.10.2020	Hexokinase (HK2)	Glycosomal ± glycolysis/gluconeogenesis	−1.42	−1.36	1.36
Tb927.9.12550	Glycerol kinase, glycosomal (glk1)	Glycosomal ± glycolysis/gluconeogenesis	−1.34	−3.38	NA
Tb927.9.12570	Glycerol kinase, glycosomal (glk1)	Glycosomal ± Glycolysis/Gluconeogenesis	−1.35	−2.97	NA
Tb927.9.12610	Glycerol kinase, glycosomal (glk1)	Glycosomal ± glycolysis/gluconeogenesis	−1.29	−2.96	NA
Tb927.9.12630	Glycerol kinase, glycosomal (glk1)	Glycosomal ± glycolysis/gluconeogenesis	−1.32	−2.95	NA
Tb927.5.4200	Histone H4, putative	Histone	−1.35	−14.81	NA
Tb927.5.4250	Histone H4, putative	Histone	−1.33	Inf Down	NA
Tb927.11.900	Isocitrate dehydrogenase, putative	Mitochondrial ± citrate cycle	−1.34	1.10	1.06
Tb927.11.6330	6-Phosphogluconolactonase	Pentose phosphate pathway	−1.37	−1.72	−1.14
Tb927.3.2960	Inosine-adenosine-guanosine-nucleosidehydrolase	Purine metabolism ± transport	−1.81	−6.55	−2.06
Tb927.2.6240	Adenosine transporter 2 (TbNT5)	Purine metabolism ± transport	−1.34	−2.75	−1.12
Tb927.6.820	Pumilio RNA binding protein, putative (PUF4)	RNA binding protein	−1.30	−1.32	−1.21
Tb927.10.12760	Zinc finger protein family member, putative (ZC3H36)	RNA binding protein	−1.40	−3.16	1.32
Tb927.9.11760	DNA repair protein RAD2, putative	Other	−1.30	1.38	1.07
Tb927.7.6220	Protein kinase, putative	Other	−1.38	−2.29	1.11
Tb927.9.10170	Predicted zinc finger protein (homolog of *Tbg* APC11)^*e*^	Other	−1.33	−1.08	1.12

a After 24 h of DDD00015314 treatment, 95 genes were upregulated, and 73 genes were downregulated greater than 1.2- fold. Further, the CAT reporter gene coupled to the PAD1 3′ UTR showed a 1.47-fold increase in mRNA expression, similar to the 1.44-fold increase in PAD1 mRNA expression (highlighted in boldface). Here, hypothetical, VSG, and retrotransposon hot spot protein genes have been removed for clarity. A full list of genes (with associated statistics) up- and downregulated after DDD00015314 treatment is provided in Data Set S2 in the supplemental material. Product assignments were based on annotations and comments at TriTrypDB.org.

b ESAG, expression site-associated gene; PAD, protein associated with differentiation; CoA, coenzyme A; PSSA, procyclic form surface phosphoprotein; LRRP, leucine-rich repeat protein; PGKC, phosphoglycerate kinase.

c ±, with or without.

d The absolute fold change in transcript abundance during complete differentiation from slender to stumpy forms as observed in two independent analyses (32, 33) is also shown for each gene. In Capewell et al. (32) PAD1 was not distinguishable from other PAD family transcripts. NA, not available.

e *Tbg*, Trypanosoma brucei gambiense.

As expected, complete differentiation from slender to stumpy forms did not occur upon DDD00015314 treatment; differentiation to stumpy forms is associated with large-scale changes in gene expression (32, 33, 35, 36), whereas DDD00015314 treatment caused changes in mRNA expression only for a limited number of genes. Nonetheless, of the changes that did occur, many were associated with transcript changes known to occur during stumpy formation, and clear expression trends were observed with functionally coordinated transcripts being coregulated (Table 1 and Fig. 6B). Further, while these changes appear modest (i.e., <2-fold change between treated and untreated cells), the changes observed during complete physiological differentiation from slender to stumpy forms are also in this range (Table 1, columns 5 and 6; see also Table S1 in the supplemental material) (32, 33). As the cell line used for this transcript analysis expressed the CAT reporter gene coupled to the PAD1 3′ UTR, it was also possible to monitor the mRNA expression levels of the CAT reporter gene as well as the endogenous PAD1 gene itself. This revealed good agreement between the two transcripts, with a 1.47-fold upregulation of CAT mRNA in response to DDD00015314 treatment and a 1.44-fold increase in PAD1 mRNA expression (Table 1). A corresponding upregulation of the PAD1 protein was not expected since the expression of this protein appears to be restricted in monomorphic cells (see Fig. S1 in the supplemental material). Supporting specificity of the observed changes, the mRNA expression level of the constitutively expressed GUS reporter in the same cell lines was unchanged compared to that in untreated cells (1.06-fold difference).

Analyzing the regulated changes after exposure of cells to DDD00015314 demonstrated the elevation of several stumpy-enriched transcripts. These included PAD2, known to be upregulated in stumpy and procyclic forms (23) (upregulated 1.37-fold), and two ESAG9 genes (Tb927.7.170 and Tb927.9.7340), also known to be enriched in stumpy forms (37). Similarly, the mRNA levels of two procyclic form surface proteins (Tb927.6.150 and Tb927.10.11220) were also increased, consistent with their elevation in stumpy forms in preparation for expression upon differentiation to procyclic forms in the tsetse midgut. Indeed, GPEET is the predominant surface protein found on early procyclic forms (38), and multiple genome-wide analyses have shown that mRNA expression of Tb927.6.150, a GPEET 2 precursor, is upregulated in stumpy forms compared to levels in slender forms (33, 35, 36). Similarly, Tb927.10.11220, or PSSA-2, is a procyclic-form phosphoprotein that has been shown by Northern blotting to have greatly increased mRNA expression in procyclic forms compared to bloodstream slender forms (39), which is preceded by its upregulation also in stumpy forms compared to slender forms (33, 35, 36).

Most notably, a number of genes associated with metabolism were seen to have altered mRNA expression after DDD00015314 treatment (Fig. 6B). Slender bloodstream form trypanosomes have a repressed mitochondrion and generate ATP solely from glucose through glycolysis, with most of this pathway localized within the glycosomes (40–42). Stumpy forms, however, have a partially elaborated mitochondrion in preparation for differentiation to the procyclic form (43), which has a fully developed mitochondrion and can metabolize amino acids, particularly proline, as well as glucose. Notably, DDD00015314 treatment caused upregulation of 10 mitochondrial proteins and five proteins involved in amino acid metabolism or transport, including proline dehydrogenase and glutamate dehydrogenase (Tb927.7.120 and Tb927.9.5900, 1.30-fold and 1.32-fold upregulated, respectively). Conversely, there was a downregulation of 10 glucose transporter gene transcripts and 13 glycosomal proteins, including proteins that play a role in glycolysis (Table 1 and Fig. 6B). Thus, DDD00015314 treatment appears to cause a shift in the expression of several metabolic genes indicative of early steps in differentiation in monomorphic cells.

### DDD00015314 promotes the development to stumpy forms in pleomorphic parasites.

**FIG 7:**
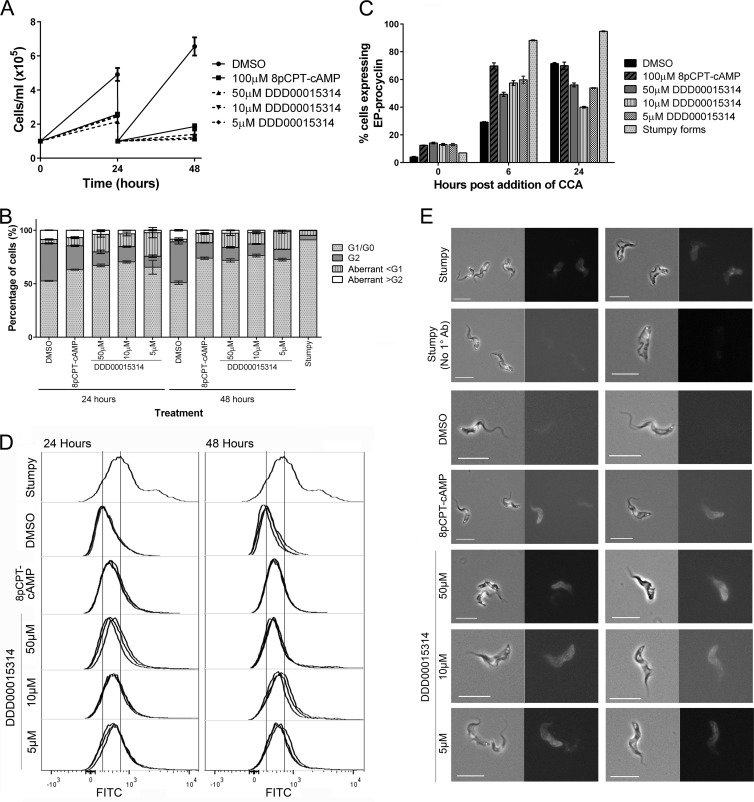
Compound DDD00015314 causes a stumpy-like phenotype in pleomorphic bloodstream form cells. AnTat1.1 90:13 cells were treated with 50 μM, 10 μM, or 5 μM DDD00015314. Negative-control cells were treated with 0.5% (vol/vol) DMSO; positive-control cells were treated with 100 μM 8-pCPT-cAMP. (A) Population growth was monitored at 24 and 48 h. Cells treated with DDD00015314 or 8-pCPT-cAMP showed reduced growth compared to DMSO-treated controls. (B) Treatment with DDD00015314 or 8-pCPT-cAMP caused an accumulation of cells in G_1_/G_0_ after 24 and 48 h, as determined by flow cytometry of DAPI-stained populations. (C) After 24 h of treatment, cells were induced to differentiate to procyclic forms by the addition of 6 mM *cis*-aconitate and a temperature change from 37°C to 27°C. Cells treated with DDD00015314 or 8-pCPT-cAMP differentiated more efficiently than DMSO-treated cells by 6 h postinduction, as determined by expression of EP procyclin. At 24 h however, cells treated with DDD00015314 had differentiated less well than DMSO-treated cells. CCA, *cis*-aconitate. (D) Treated cells were fixed and stained for PAD1 protein expression and analyzed by flow cytometry. Cells treated with DDD00015314 or 8-pCPT-cAMP showed upregulation of PAD1 protein expression compared to negative controls after 24 and 48 h although to a lesser degree than that observed from a stumpy form positive control. FITC, fluorescein isothiocyanate. (E) Immunofluorescence microscopy of cells after 24 h of treatment confirms upregulation of PAD1 protein expression in DDD00015314- or 8-pCPT-cAMP-treated cells. Two examples are shown per treatment. In each pair, the left panel shows a phase-contrast with DAPI image and the right panel shows the same cells stained for PAD1 protein. 1° Ab, primary antibody.

Given that DDD00015314 was able to induce a partial differentiation phenotype in monomorphic trypanosomes, we investigated whether this compound would have a similar, or more pronounced, effect on pleomorphic cells naturally competent for stumpy formation. Therefore, a pleomorphic *T. brucei brucei* AnTat 1.1 cell line was treated with 50, 10, or 5 μM DDD00015314 for up to 48 h. At all concentrations tested, DDD00015314 caused decreased population growth (Fig. 7A) and accumulation in G_1_/G_0_ (Fig. 7B) equivalent to that induced by 100 μM 8-pCPT-cAMP. Since stumpy forms have an increased capacity for differentiation to procyclic forms compared to slender forms, cells that had been compound treated for 24 h were then induced to differentiate to procyclic forms by the addition of 6 mM *cis*-aconitate and a temperature reduction to 27°C. Figure 7C shows that DDD00015314-treated cells showed an increased level of differentiation after 6 h, with a mean differentiation after 50 μM, 10 μM, and 5 μM DDD00015314 treatment of 49.3%, 57.4%, and 59.7%, respectively, compared to 29.2% in cells treated with DMSO alone (Fig. 7C). This was not, however, as effective as 8-pCPT-cAMP exposure, nor did DDD00015314 treatment produce the levels of differentiation observed in *in vivo*-derived stumpy forms, which had a mean differentiation of 69.8% and 88.2%, respectively. Moreover, by 24 h postinduction of differentiation, DMSO-treated controls exhibited a higher level of EP procyclin-positive parasites than DDD00015314-treated cells, perhaps indicating that the treatment allows cells to embark on the differentiation pathway but not sustain a proliferative procyclic population.

**FIG 8:**
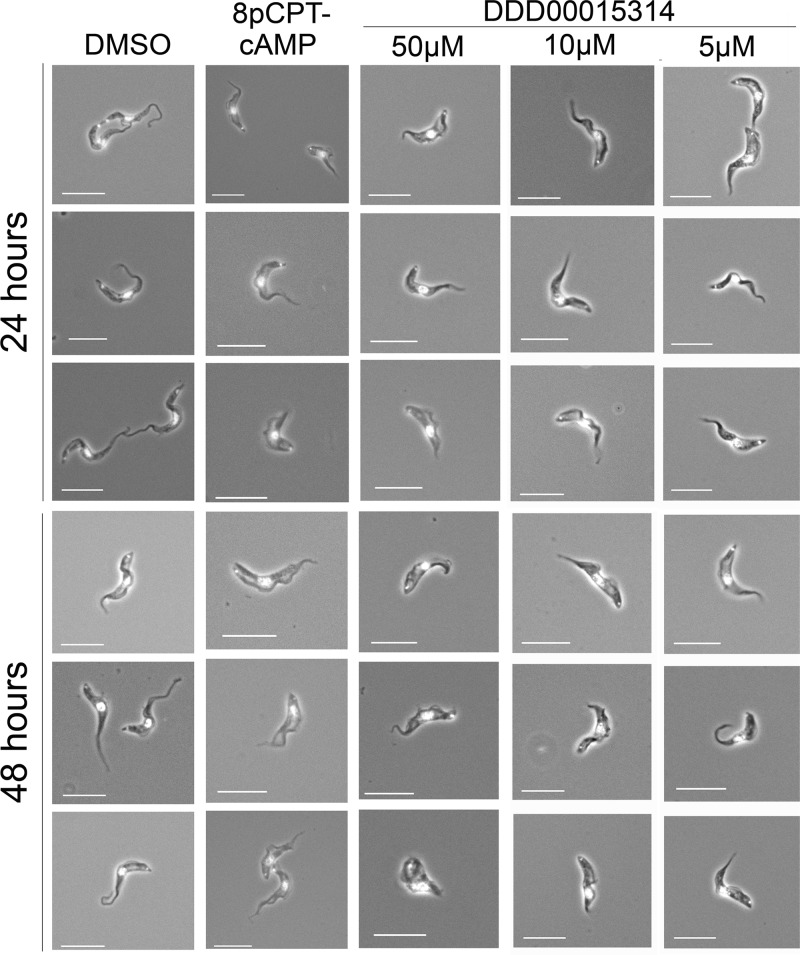
Compound DDD00015314 causes a stumpy-like morphology in pleomorphic bloodstream form cells. Microscope images of treated cells reveal that DDD00015314 or 8-pCPT-cAMP treatment induces a stumpy-like phenotype after 24 h. This morphology remains apparent in cells treated with 5 or 10 μM DDD00015314 or with 8-pCPT-cAMP after 48 h, but many cells treated with 50 μM DDD00015314 have an aberrant morphology after 48 h.

As a final measure of the development to stumpy forms induced by DDD00015314, we analyzed the expression of the stumpy-specific marker protein, PAD1, by both flow cytometry (Fig. 7D) and immunofluorescence microscopy (Fig. 7E). With both methods, an increase in PAD1 protein expression in the population was observed after treatment with 8-pCPT-cAMP and at all concentrations of DDD00015314 tested. Further, microscopy revealed the presence of cells with intermediate and stumpy-like morphology after 24 and 48 h of treatment with either 8-pCPT-cAMP or DDD00015314 (Fig. 7E and Fig. 8). After 48 h of 50 μM DDD00015314 treatment, however, cells began to look misshapen. Thus, although the effects of DDD00015314 are modest in monomorphic cells, treatment of pleomorphic cells with this compound has substantial effects, generating an accumulation in G_1_/G_0_, expression of the PAD1 protein, increased capacity for the initiation of differentiation to procyclic forms, and morphological changes associated with stumpy formation.

In conclusion, from a pilot screen of over 6,000 compounds from a kinase-targeted library, a compound has been identified that is able to specifically activate a reporter for stumpy-enriched gene expression in monomorphic cell lines and induce a stumpy-like phenotype in pleomorphic cell lines, as characterized by a number of diagnostic assays. Moreover, an analysis of the transcriptome of treated monomorphic cells revealed a modest but consistent elevation of a number of transcripts known to be regulated during the development to stumpy forms *in vivo*. This study, therefore, validates high-throughput screening as an approach to identify developmental regulators in trypanosome parasites. Moreover, a novel tool compound applicable for dissection of components of this developmental pathway is identified.

## DISCUSSION

Whole-cell-based high-throughput screening has been successfully utilized in Trypanosoma brucei research in the search for compounds with trypanocidal or trypanostatic activity, with the aim of identifying new lead compounds for the development of novel therapeutics against African trypanosomiasis (4–10). Here, we explored the potential for using a similar screening method for the identification of compounds able to induce other trypanosome phenotypes, namely, differentiation between life stages, providing possible tool compounds of value in research, as well as therapeutics exploiting the irreversible proliferation arrest associated with the transition to stumpy forms. The fluorescence-based approach developed was a rapid, inexpensive, and simple assay able to be carried out in 96-well plate format, and autofluorescent library compounds could be rapidly excluded in a cell-free assay pre- or postscreening to identify cytologically relevant hits. As such, over 6,000 compounds could be screened on fewer than 100 plates, requiring less than 1 liter of trypanosome culture. Smaller-scale (and hence lower-cost) validation screening using a non-fluorescence-based reporter that also allowed reporter-specific effects to be distinguished from general gene expression effects was then exploited to unambiguously identify compounds able to drive the developmental response under test.

From a starting point of 6,764 compounds, this screening method successfully identified three compounds able to induce upregulation of PAD1 reporter gene expression as a proxy for stumpy formation, and these were validated through follow-up analysis. This confirmed the efficacy of the approach for detecting specific cytological phenotypes distinct from generalized cytostatic and cytotoxic effects likely to be elicited by a much broader range of hit compounds. Two of the three hit compounds were structurally very similar; the identification of both of these molecules from a large compound set illustrates the consistency and reproducibility of the method. Follow-up analysis confirmed that the increase in PAD1 reporter gene expression induced by one of these compounds (compound DDD00070762) was consistent and reproducible. However, it did not represent a specific physiological progression to a stumpy or stumpy-like form since the constitutive reporter employed in the secondary screen exhibited similar (though inconsistent) elevation and since the exposed cells rapidly became multinucleated. Hence, these compounds may target one or more processes in the cell that generate phenotypes distinct from, or in addition to, a specific developmental response. In contrast, the third compound identified, DDD00015314, did cause specific upregulation of both PAD1 reporter gene activity and endogenous PAD1 mRNA levels, without activation of the constitutive reporter in monomorphic lines. Moreover, upon treatment with DDD00015314, the cells underwent a change in expression of a restricted set of genes, many of which are also elevated upon stumpy formation *in vivo*. In particular, several genes relating to energy metabolism were elevated (mitochondrial genes and enzymes involved in proline metabolism), whereas the mRNA levels of a number of genes involved in glucose transport and glycolysis were downregulated. These responses were limited in extent (albeit similar in scale to those seen in the natural developmental process) but reproducible and indicative of a progression toward the stumpy phenotype in the monomorphic lines used in the high-throughput screen. Treatment of pleomorphic cells with the same compound had a more pronounced effect, even at lower concentrations: cells displayed a stumpy-like phenotype with an accumulation of cells in G_1_/G_0_, expression of PAD1 protein, and the presence of cells with an intermediate or stumpy morphology within the population. Although the response was not entirely physiological since the elevation of EP procyclin expression at 6 h after exposure to *cis*-aconitate was diminished after 24 h, the data indicate that compound DDD00015314 is able to induce partial stumpy formation or activate/inhibit some components of the stumpy formation pathway.

Cell-permeable hydrolysable cAMP and AMP analogues have been utilized for the induction of some, albeit not all, stumpy characteristics in monomorphic cells, providing reagents to analyze some events of stumpy formation. Recently, a whole-genome RNAi screen was designed to identify genes required for responsiveness to cAMP and AMP analogues in monomorphs, resulting in the identification of multiple genes involved in physiological stumpy formation *in vivo*. These represented the first known positive inducers of stumpy formation encompassing molecules from throughout the signaling cascade, including kinases, phosphatases, and at least one predicted RNA binding protein (26). Clearly, the outputs from high-throughput small-compound screens such as the screen described here could provide reagents to further dissect this pathway or other uncharacterized signaling pathways, for example, by assisting in the ordering of components following further genome-wide RNAi screens. Indeed, using multiple and distinct chemical inducers of a full or partial stumpy-like phenotype would allow iterative RNAi screens, with resistance phenotypes being observed only for molecules falling downstream of where the compounds act on the signaling pathway. As compound DDD00015314 appears to induce partial differentiation toward stumpy formation, it could be targeting only some aspects of the SIF signaling pathway, such that a whole-genome RNAi screen with this compound could identify genes involved in a specific branch of a stumpy formation cascade as well as those shared with the response to cell-permeable cAMP/AMP. Currently, the limited availability of compound DDD00015314 and its expense preclude such analysis with a monomorphic RNAi library (44); however, the enhanced sensitivity of pleomorphic cells could permit this analysis if RNAi libraries could be generated at sufficient coverage in these cell lines. Alternatively, DDD00015314 could be chemically refined to increase its potency and specificity in order to potentially achieve activity at lower concentrations. This provides a starting point, and proof of principle, for the development of further tools for the study of trypanosome differentiation and developmental gene regulation.

Compounds driving complete or incomplete stumpy formation also offer potential as a complementary or adjunctive approach to trypanocidal therapeutics, generating a reduction of parasite virulence or transmission between hosts. Hence, accelerated differentiation of slender forms to stumpy forms within the bloodstream could reduce transmission in epidemic situations by reducing the density of stumpy forms below that needed for effective tsetse passage. Perhaps more importantly, where a complete differentiation of parasites into stumpy forms could be achieved in the bloodstream, a trypanostatic response (stumpy formation) would inevitably lead to a trypanocidal outcome through the action of the immune response (45), clearing the infection. Unlike conventional therapies, this approach could also be evolution resistant since emergent resistant parasites, while more virulent in the originating host, would have reduced transmissibility through their reduced rate of stumpy formation, preventing the propagation of resistance in a population.

## Supplementary Material

Supplemental material
