# Supplementary material for: High-Throughput Chemical Screening for Antivirulence Developmental Phenotypes in Trypanosoma brucei
Source: Eukaryot Cell. 2014 Mar;13(3):412–26. doi: 10.1128/EC.00335-13 (PMC3957582; doi:10.1128/EC.00335-13)
Supplement: Supplemental material [file supp_13_3_412__index.html]

Supplemental material 

# High-Throughput Chemical Screening for Antivirulence Developmental Phenotypes in Trypanosoma brucei

## Supplemental material

**Files in this Data Supplement:**

- Supplemental file 1 -

  Fig. S1 and S2 and Table S1.

  PDF, 2.9M
- Supplemental file 2 -

  Compound screen data (Data Set S1).

  XLS, 165K
- Supplemental file 3 -

  Expression data set (Data Set S2).

  XLSX, 2.8M
