# Supplementary material for: High-Throughput Chemical Screening for Antivirulence Developmental Phenotypes in Trypanosoma brucei
Source: Eukaryot Cell. 2014 Mar;13(3):412–26. doi: 10.1128/EC.00335-13 (PMC3957582; doi:10.1128/EC.00335-13)
Supplement: Supplemental material [file EC.00335-13_zek999094247so1.pdf]

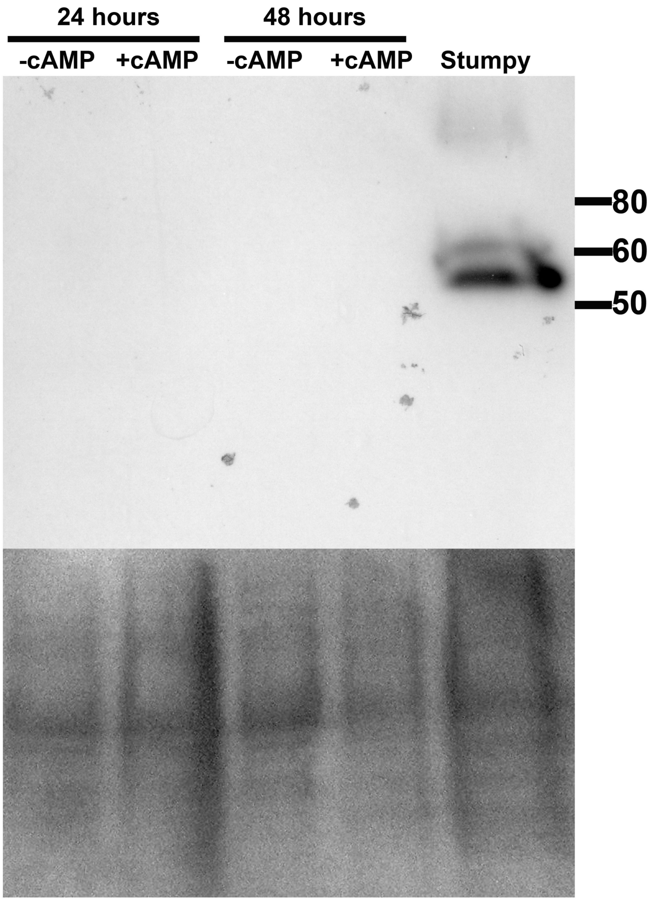


**Supplementary Figure 1: cAMP treatment does not cause upregulation of PAD1 protein expression.** 427 GUS-PAD1 3'UTR cells were treated with 100µM 8pCPT-cAMP for 24 and 48 hours. Protein was harvested and analysed via western blot for presence of the PAD1 protein. Stumpy cells from the pleomorphic AnTat1.1 cell line were used as a positive control. Lower panel shows Ponceau staining as loading control.

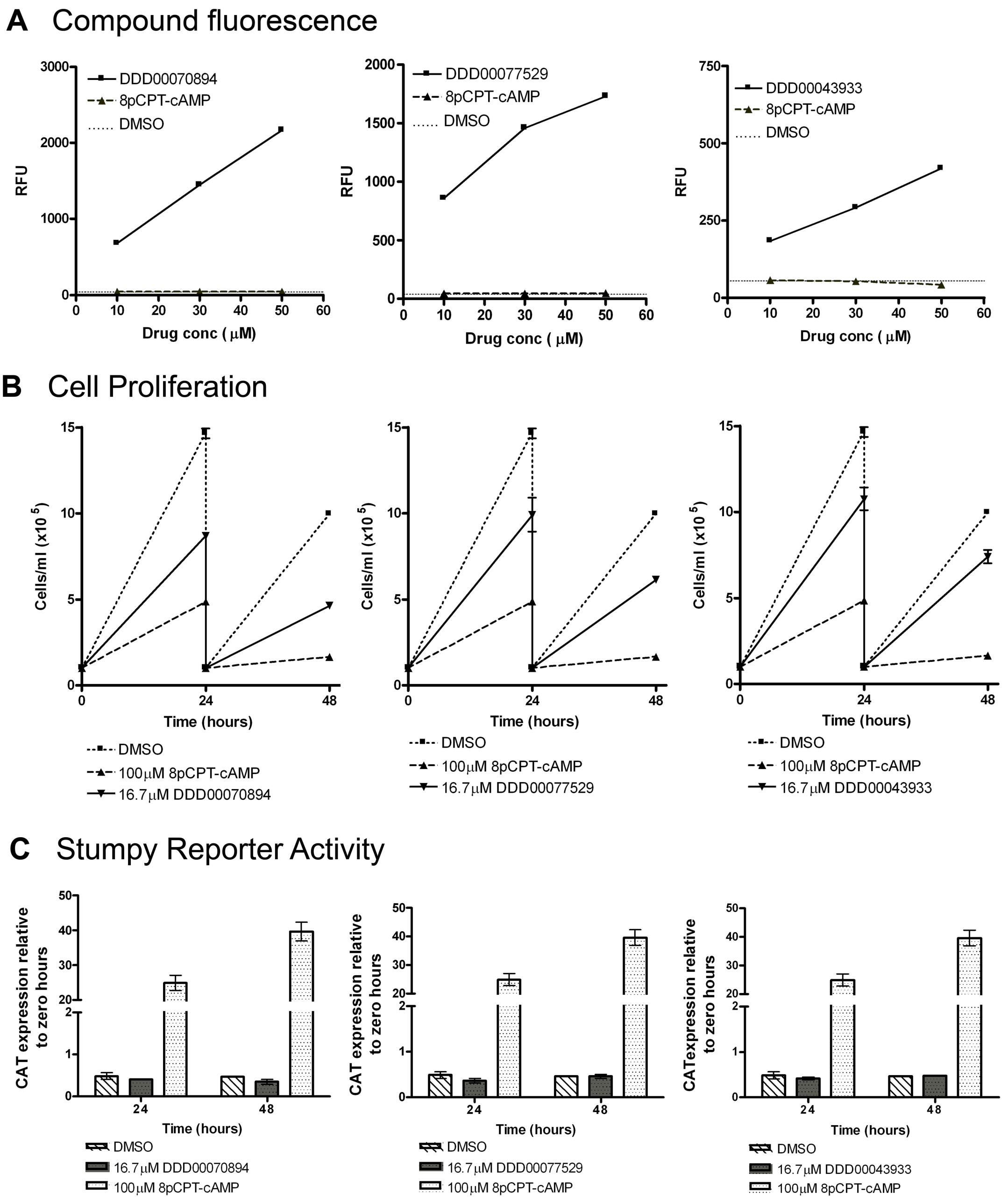


**Supplementary Figure 2: Analysis of three fluorescent compounds identified through screening.** (A) Compounds DDD00070894, DDD00077529 and DDD00043933 were shown to be fluorescent in HMI-9 at λex 340nm, λem 460nm at 5.6μM, 16.7μM and 50μM, with 8pCPT-cAMP included as a negative control. Dashed line represents 0.5% v/v DMSO in HMI-9 alone. (B) 427 CAT-PAD1 3'UTR GUS-Const 3'UTR cells were treated with 50μM of each compound and population growth was monitored at 24 and 48 hours. Cells treated with each of the three compounds showed reduced growth compared to DMSO treated

controls. (C) CAT reporter gene expression, under the control of the PAD1 3'UTR, did not increase after 24 and 48 hours treatment with any of the three compounds. Negative control cells were treated with 0.5% v/v DMSO, positive control cells were treated with 100µM 8pCPT-cAMP. Error bars represent s.e.m., n=3.

| | This study | Capewell et al., 2013 | Jensen et al., 2009 |
|---|---|---|---|
| Number of genes included | 8723 | 8290 | 7492 |
| Mean increase | 1.07 | 1.69 | 1.2 |
| Mean decrease | 1.07 | 1.73 | 0.828 |
| Mean increase >1.25 fold | 1.39 (155) | 2.36 (2993) | 1.45 (1208) |
| Mean decrease >1.25 fold | 1.49 (139) | 2.47 (2854) | 1.48 (1089) |
| Median increase >1.25 fold | 1.33 (155) | 1.74 (2993) | 1.37 (1208) |
| Median decrease >1.25 fold | 1.35 (139) | 1.78 (2854) | 1.4 (1089) |

**Supplementary Table 1: A summary of the observed changes in transcript abundance observed in this study after DDD00015314 treatment in comparison to the changes observed during full differentiation from slender to stumpy forms.** The mean and median changes in mRNA abundance during differentiation from slender to stumpy forms was calculated from datasets generated by Capewell *et al.,* 2013 and Jensen *et al.,* 2009 (1, 2). For each data set only those genes identified in both this study and Capewell *et al.,* 2013 (8290 genes) or both this study and Jensen *et al.,* 2009 (7492 genes) were included for analysis.

**References**

1. **Capewell P, Monk S, Ivens A, MacGregor P, Fenn K, Walrad P, Bringaud F, Smith T, Matthews KR.** 2013. Regulation of Trypanosoma brucei total and polysomal mRNA during development within its mammalian host. PLoS One **8(6):** e67069.

2. **Jensen BC, Sivam D, Kifer CT, Myler PJ, Parsons M.** 2009. Widespread variation in transcript abundance within and across developmental stages of Trypanosoma brucei. BMC genomics [electronic resource] **10:**482.
